# SIAIP position paper: provocation challenge to antibiotics and non-steroidal anti-inflammatory drugs in children

**DOI:** 10.1186/s13052-018-0589-3

**Published:** 2018-12-07

**Authors:** Carlo Caffarelli, Fabrizio Franceschini, Davide Caimmi, Francesca Mori, Lucia Diaferio, Dora Di Mauro, Carla Mastrorilli, Stefania Arasi, Simona Barni, Paolo Bottau, Silvia Caimmi, Fabio Cardinale, Pasquale Comberiati, Giuseppe Crisafulli, Lucia Liotti, Umberto Pelosi, Francesca Saretta, Gianluigi Marseglia, Marzia Duse, Francesco Paravati

**Affiliations:** 1 Clinica Pediatrica, Department of Medicine and Surgery, University of Parma, Via Gramsci 14, 43122 Parma, Italy; 2 Pediatric Unit, “G. Salesi” Children Hospital, Ancona, Italy; 3 Allergy Unit, Departement de Pneumologie et Addictologie, Hôpital Arnaud de Villeneuve, CHRU de Montpellier, Montpellier, France; 4 Allergy Unit, Department of Pediatric, Anna Meyer Children’s University Hospital, Florence, Italy; 5 Department of Paediatrics, Aldo Moro University of Bari-Giovanni XXIII Hospital, Bari, Italy; 6 Pediatric Allergy Unit, Bambino Gesù Academic Hospital, Rome, Vatican State Italy; 7 Department of Paediatrics and Neonatology, Ospedale di Imola, Azienda USL, Imola, Italy; 8 Pediatric Clinic, Department of Pediatrics, University of Pavia, Pavia, Italy; 9 Department of Pediatrics, Pediatric Hospital “Giovanni XXIII”, University of Bari, Bari, Italy; 10 Pediatric Clinic, Department of Life and Reproduction Sciences, University of Verona, Verona, Italy; 11 Allergy Unit, Department of Pediatrics, University of Messina, Messina, Italy; 12 Pediatric Unit, Civic Hospital, Senigallia, Italy; 13 Pediatric Unit, Santa Barbara Hospital, Iglesias, Italy; 14 Pediatric Unit, Palmanova Hospital, Palmanova, Italy; 15 Pediatric Clinic, University of Pavia, IRCCS Policlinico “S. Matteo” Foundation, Pavia, Italy; 16 Department of Pediatrics, Policlinico Umberto I, Sapienza University of Rome, Rome, Italy; 17 Pediatric Unit, Infant Maternal Department, Azienda Sanitaria Provinciale Crotone, Crotone, Italy

**Keywords:** Allergy, Antibiotics, Challenge, Children, Diagnosis, Drug allergy, Drug hypersensitivity reactions, Non-steroidal anti-inflammatory drugs, Pediatric

## Abstract

Drug hypersensitivity reactions (DHRs) in childhood are mainly caused by betalactam or non-betalactam antibiotics, and non-steroidal anti-inflammatory drugs (NSAIDs). Laboratory tests for identifying children who are allergic to drugs have low diagnostic accuracy and predictive value. The gold standard to diagnose DHR is represented by the drug provocation test (DPT), that aims of ascertaining the causative role of an allergen and evaluating the tolerance to the suspected drug. Different protocols through the administration of divided increasing doses have been postulated according to the type of drug and the onset of the reaction (immediate or non immediate reactions). DPT protocols differ in doses and time interval between doses. In this position paper, the Italian Pediatric Society for Allergy and Immunology provides a practical guide for provocation test to antibiotics and NSAIDs in children and adolescents.

## Introduction

Data on prevalence and incidence of drug hypersensitivity reactions (DHRs) are limited, especially in the pediatric age and varies around the world [1]. About 10% of parents report that their children are allergic to drugs [2]. In prospective studies conducted in children and adolescents, the rate of adverse drug reactions was 10.9% in hospitalized children, 1.0% in outpatients, and the hospitalizations rate for adverse drug reactions was of 1.8% [3]. Mild delayed cutaneous reactions including maculopapular rashes (20–80%) and urticaria/angioedema (20–30%) are common reactions in children. Anaphylaxis and severe cutaneous reactions are rare, although they may occur up to 10% of patients with suspected drug reactions [4]. Serum Sickness-Like Reactions (SSLRs) are less common (0.02 to 0.2% of children) and mostly related to first generation cephalosporins [5]. DHRs are classified as immediate or nonimmediate/delayed reactions. Immediate reactions typically occur within one hour after the last drug administration and they are often caused by direct mast cell activation or IgE-mediated hypersensitivity. Delayed reactions occur from 1 h after drug administration and may result from antigen-specific IgG production, complement activation or a T-cell mediated response [6–8]. Reactions occurring between 1 and 6 h after the last drug intake are called accelerate and can be caused both by an IgE-mediated and T-lymphocyte mediated response. There is an overlap between accelerate and delayed reactions. Immediate drug reactions may manifest as isolated mild symptoms (urticaria, angioedema, coughing, conjunctivitis, rhinitis, nausea, vomiting, diarrhea, abdominal pain), severe respiratory symptoms (bronchospasm, dyspnea), and rarely as anaphylaxis [9]. The most common mild cutaneous non-immediate reactions are maculopapular or morbilliform exanthema, eczema, delayed urticaria and/or angioedema [10]. They appear from 6 h to 5 days after starting a course of treatment [1, 6, 8, 11, 12]. Delayed reactions can onset at any time after the initial drug administration, from 1 h to several weeks after, and can affect individual organs or systems (nephritis, pneumoniae, haemolytic anaemia, cytopenia, hepatitis, vasculitis and serum sickness) with or without cutaneous symptoms. Delayed severe cutaneous adverse reactions (SCAR) include erythema multiforme major (EMM), generalized acute exanthematous pustulosis (AGEP), drug eruption with eosinophilia and systemic symptoms (DRESS), Stevens-Johnson syndrome (SJS) and toxic epidermal necrolysis (TEN). A history of SCAR or SSLRs contraindicates the performance of drug provocation test (DPT). However, some children with SSLR to amoxicillin who tolerated DPT [13, 14] and patients with SSLR to cefaclor who tolerated other cephalosporins are reported [14]. In childhood, antibiotics, mainly penicillins, and non-steroidal anti-inflammatory drugs (NSAIDs) are the most common causes of DHRs [2, 15–21].

Antibiotics may account for 27–85% of adverse drug reactions [22]. Prevalence of respiratory symptoms induced by NSAIDs was 1.6% in subjects aged 15–20 years [23]. Large studies on the prevalence of NSAIDs hypersensitivity reactions in children are lacking [2, 24]. In this position paper, the Italian Pediatric Society for Allergy and Immunology (Società Italiana Allergologia Immunologia Pediatrica –SIAIP) provides a practical guide for provocation test to antibiotics and NSAIDs in children and adolescents.

## Drug provocation test in children

A drug provocation test (DPT) is the gold standard to diagnose DHR with specific indications, limitations and contraindications that are summarised in Table 1 [6, 25]. The aim of DPT is to evaluate the tolerance to the suspected drug and when negative, to reassure parents that the drug can be used safely. False-negative provocation tests can be due to low dosage, short duration or lack of cofactors (e.g. concomitant infections). False positive provocation tests can be due to elicitation of subjective symptoms only.

**Table 1:** Precautions, indications and contraindications of DPT

Contraindications	• SCAR, Serum Sickness-Like Reactions, internal organ or system involvement. • Availability of alternative drugs, the drug will be probably not used in the future. • Suggestive clinical history, positive allergy tests. • Active disease such as uncontrolled asthma, urticaria, intercurrent infections, severe allergic rhinitis. Conditions that may affect treatment of allergic reactions, such as cardiovascular disease, treatment with β-blockers.
Precautions	• The risk/benefit balance must be discussed with patient and caregivers in children with anaphylaxis.
Indications	• Mild reactions. • Suggestive clinical history with negative or unavailable allergy tests. • Clinical history not suggestive and/or non-specific symptoms. • To exclude cross-reactivity with related drugs in subjects with known drug reaction.

A single or double-blind DPT using as placebo, inert substances such as talcum powder or methylcellulose inside opaque jelly capsules is necessary in patients reporting only subjective or doubtful symptoms and in those with a history of multiple drug intolerance [26]. DPT with the culprit drug is not recommended in patients with history of severe immediate or delayed reactions (Table 1).

DPT should always be supervised by personnel trained to promptly recognize and treat acute allergic reactions including anaphylaxis [27, 28]. It should be conducted in a setting with adequate facilities for continuous monitoring of the patient condition and with emergency treatment available. Patients must be healthy. Concurrent diseases may interfere with interpretation of DPT and increase the risk of a serious reaction. (Table 1). Medications that may affect the DPT such as oral antihistamines, systemic corticosteroids, short-acting beta2-agonists, ipratropium bromide should be discontinued [25, 27]. Administration of drugs for asthma control such as inhaled corticosteroids, long-acting beta2-agonists, leukotriene antagonists, chromones and theophylline may be continued. A fasting period of 2–4 h prior to the DPT may be considered. At baseline, vital signs (respiratory rate, heart rate, blood pressure) should be obtained and physical findings should be recorded to help as reference. Insertion of an intravenous line should be considered. Lung function tests may be performed prior to DPT when necessary, especially in asthmatics [27, 28].

## Betalactams

Hypersensitivity to betalactams, mostly amoxicillin-clavulanic acid, is suspected in 1.5–12% of children, while third-generation cephalosporins are less frequently involved [29]. A study of 2,375,424 children and adults in Southern California, showed that prevalence of allergy to penicillin was 7.9% [30]. The diagnostic work-up for betalactams usually shows that the prevalence and incidence of hypersensitivity reactions to betalactams are overestimated [2]. A high negative predictive value of 94% of beta lactam DPT has been found [2, 31]. In a large study involving both children and adults [31] most of the reactions elicited by DPT were mild, non-immediate and cutaneous (urticaria or rash).

The administration of alternative betalactam drugs in people who have already experienced an allergic reaction to a drug of the same class is a key issue. Cross-reactions between penicillins and cephalosporins is reported in less than 10% of patients allergic to penicillins and mainly depends on the molecular structure of the side chain placed in position − 7 of the betalactam ring [32, 33]. The probability of cross-reaction decreases from the first generation cephalosporins to the third-generation that appear to be at low risk of cross-reactions [34–38]. Reactions to penicillins in patients allergic to cephalosporins have been sparsely investigated. Less than 20% of cephalosporin allergic patients showed positive skin tests to penicillins, mostly due to cross reactivity with cephalosporins with a similar side chain [29, 39]. DPT helps also to exclude cross-reactivity between drugs belonging to the same class, as it happens for cephalosporins. Some reactions are directed towards specific determinants of the lateral chain R1 of cephalosporins. Therefore, allergic patients can tolerate cephalosporins with a different R1 side chain [40]. Cross-reactivity between penicillins or cephalosporins with other betalactams is rare [41].

It is recommended to perform the diagnostic work-up for betalactams reactions 1–6 months after the allergic reaction because afterwards the tests may become negative. In vivo tests, skin prick tests (SPT) and intradermal skin test (ID) to minor determinant mixture (MDM), benzyl penicilloyl-polylysine (PPL), ampicillin, benzylpenicillin, amoxicillin, cephalosporins are validated and their negative predictive value is high for immediate reactions [42]. SPT and ID for clavulanic acid are reported to be positive in 30% of adults who reacted to amoxiclavulanate [43]. Their diagnostic accuracy is limited by the difficulty of having a stable solution of clavulanic acid suitable for in vivo or in vitro testing [44]. Detection of serum-specific IgE in vitro is available for penicillin G, penicillin V, amoxicillin, ampicillin and cefaclor. The sensitivity of these tests depends on the antigen, on the time interval between reaction and IgE measurement and on the clinical reaction. When IgE tests are negative, a DPT should be performed according to the criteria displayed in Table 1. In case of immediate reactions to beta-lactams that occurred more than 6 months before a negative DPT, another drug challenge may be performed after 2–3 weeks, to exclude a possible re-sensitization. Performing SPT/IgE tests before a re-challenge, may be considered. The rate of positive rechallenge varies from 2% in children to 23% in studies on adults and children [45–47]. In non-immediate reactions, delayed-reading ID, SPT and patch tests can be used with low sensitivity, as reported in the diagnosis of mild delayed cutaneous reactions [2, 48–50]. Therefore, in mild cutaneous delayed reactions, it is suggested to perform only DPT [2, 48, 49] because of its high negative predictive value [50, 51], without skin testing and serum IgE measurement.

### DPT in immediate reactions

There is no standardized protocol for DPT in children with immediate reactions or when the time interval between reaction and exposure to the drug is unclear. Divided incremental doses of the single therapeutic dose are usually administered in a multistep provocation test. Determining the initial dose depends mainly on clinical history and the probability of true drug allergy [26]. It has been reported that patients is associated with a lower risk for development of a severe acute reaction to the drug may be challenged with a higher starting dose [26]. DPT is usually started with 1/100 or 1/10 of the single therapeutic dose calculated for the weight and the age of the child. The European Network on Drug allergy (ENDA) Task Force [2] recommends starting at 1/10 of the dose and then administering half and the full dose. In the event of severe reactions, the initial dose is debated. Some European authors suggest starting with 1:10000 or 1:1000 of the maximum therapeutic dose and then administering increasing doses until the maximum single therapeutic dose is reached [49]. A recent study by Chiriac and colleagues [52] have suggested a new protocol for DPT to beta-lactams based on the analysis of 182 positive challenge tests. They administered four doses, containing 5-15-30-50% of the single therapeutic dose, with additional steps in case of anaphylaxis. Therefore, a 1 day protocol was appropriate for immediate mild reaction or delayed mild reaction. Patients should be kept under surveillance for 2 h after administration of the last dose and for additional 48 h after discharge. The time interval between doses should be based on clinical history and it can vary from 20 min to 1 week. Generally, an interval of 20 to 60 min between each dose is considered appropriate for patients with a history of immediate reactions. Longer time intervals have been suggested for non-immediate reactions [29]. A protocol with 3 or more steps has not been approved in the United States suspecting that an unintentional desensitization can be induced [2]. Furthermore, a DPT in three or more steps are more expensive and time-consuming while severity and frequency of reactions are comparable to those occurring in a single-dose or two-dose provocation tests [26]. If an immediate hypersensitivity reaction occurs during DPT and alternative drugs are unavailable, a desensitization protocol should be considered.

### DPT in delayed reactions

The methods of provocation test in case of delayed reactions are widely debated and an international consensus is still lacking. In Europe, DPT aims to confirm or exclude the diagnosis of hypersensitivity to beta-lactams while in United States, it aims to exclude the diagnosis of hypersensitivity in low-risk cases [53]. Therefore, the protocols of DPT in United States and Europe are different. There is difference of opinion on the administration of divided increasing doses or a single dose [54]. Furthermore, although it is recognized that administering the daily therapeutic dose for 3 to 7 consecutive days reduces the risk for future reactions, members of the ENDA Task Force [2] and some American authors [54] believe that exposure to a single therapeutic dose is enough to reach the diagnosis in most of the cases [2]. However, the rate of subjects with positive DPT was higher in patients who continued to take the drug for at least 3–5 days at home than in those who did not [15, 55–59]. Reactions reported after an extended DPT at home have been found to be mild [60]. More studies are necessary to assess risks/benefits of a prolonged DPT in terms of sensitivity, cost and adherence.

#### Betalactam provocation test protocol

- Immediate reactions (Table 2). A DPT involves administering 1/10 or 1/100 of the single therapeutic dose then 2/10 and 7/10 every 30 min with a minimum of two-hours surveillance after the last dose. Administration of an additional single full dose of the drug the same day or the next day may be considered [2]. Patient should be contacted 48 h after discharge, because the elimination of the drug requires this duration, and delayed reactions due to anamnestic errors may occur [50].

**Table 2:** Betalactam provocation test

Symptoms	Procedures
Immediate reactions with negative skin tests	Graded challenge 1/10 TD (1/100 TD or 1/1000 TD when risk is high, e.g. severe asthma) then 2/10 TD and 7/10 TD every 30 min 2 h observation after last dose
Delayed reactions	Graded challenge as DPT for immediate reactions, or a full-dose challenge. The daily therapeutic dose is given at home for a minimum of 5 days up to 7 days.
Positive skin tests Anaphylaxis	Desensitization

*TD* therapeutic dose

- Delayed reactions. Incremental doses as those administered in DPT for immediate reactions, or a single dose are given on the first day under physician supervision [2]. The test is continued at home and the therapeutic dose is taken once or twice a day for a minimum of 5 days (up to 7 days) to elicit the T cell response [55]. Alternatively, a single therapeutic dose is given at the office, with a surveillance period of 2–5 days at home. The patient is asked to contact the physician to communicate the outcome of the test [61]. If any reaction occurs at home, the patient must be re-examined.

## Non-betalactam antibiotics

The prevalence of allergic reactions to non-betalactam antibiotics (NBLA) is estimated to be 1–3% of the general population and represents about 10% of DHRs in children [62]. Viral infections can provoke skin eruptions such as macular exanthemas that is also the most common symptom of allergic reactions to NBLAs. Therefore, it is difficult to differentiate DHRs from skin symptoms due to infections.

The main classes involved in DHRs in children are sulphonamides, macrolides, glycopeptides, aminoglycosides and quinolones. Reactions to tetracycline, metronidazole, nitrofurantoin antituberculosis drugs have not been associated with an immunologic mechanism and the diagnostic value of allergy tests is unclear [1, 63].

We summarized below the current knowledge on hypersensitivity reactions to specific NBLA although specific studies are scarce.

### Sulphonamides

It is recommended to perform the diagnostic work-up for sulphonamides within 1–6 months of the reaction [64]. In vivo test may be useful: SPT and immediate-reading ID for IgE-mediated reactions and delayed-reading ID for delayed reactions. Sensitivity of these tests is low, but the specificity is high [63]. DPT represents the diagnostic gold standard and the most required test in HIV + patients who often need prolonged therapies with this drug, not easily replaceable, for preventing opportunistic infections. In case of mild or moderate non immediate reactions (without mucosal signs or systemic symptoms) different strategies have been proposed. It is possible to continue cotrimoxazole administration at the same doses (treat through) or to discontinue the drug over a few months, usually 6 months, and then cotrimoxazole could be resumed after a graded challenge or a “desensitization” protocol [62]. A meta-analysis involving 268 adults with HIV infection and mild or moderate hypersensitivity reactions to cotrimoxazole found that the desensitization protocol was the most beneficial for preventing severe skin reactions, when it is performed after 6 months of drug discontinuation [65].

### Macrolides

Hypersensitivity reactions to macrolides are relatively uncommon (0.4 to 3% of treatments) [66]. Diagnostic workup for macrolides is hampered by the poor standardization of skin tests as well as by lack of accurate in vitro tests. Few studies, most of which in adult population, report a rate of positive skin tests for macrolides ranging from 28 to 43%. The sensitivity and specificity of IDs to clarithromycin at the concentration of 0.5 mg/ml are reported to be 75 and 90%, respectively [67]. In children, little data exists on non-irritant concentrations [68]. Therefore, a positive skin tests to macrolides is open to ambiguous interpretation [69]. There is also limited evidence on the usefulness of patch tests and delayed-reading IDs [67]. Thus, DPT is the only reliable diagnostic test [70], even in the absence of standardized protocol specific for macrolides. Macrolides can be administered orally or iv, but the oral route is considered safer in case of immediate reactions. The most common method for performing DPT is the graded challenge. Patients with a history of delayed reactions should continue to take the drug at therapeutic doses for at least 5 days [71].

### Glycopeptides

There are no validated in vitro tests for investigating allergy to glycopeptide. In patients with suggestive clinical history, positive immediate-reading IDs (0.1 mg/ml or lower) may identify immediate hypersensitivity reactions, and positive patch tests (concentration 0.005%) delayed hypersensitivity reactions [63]. In patients with history of mild reaction, a graded challenge should be performed [72]. Despite its chemical affinity, no cases of Red Man Syndrome and very few cases of allergic reactions were reported with teicoplanin [73] in children with previous reactions to vancomycin. Thus, in these children, teicoplanin might be administered even if there is limited evidence that is tolerated [63]. When possible, an alternative drug should be used or a desensitization protocol should be performed.

### Aminoglycosides

Although anecdotal cases of positive SPT to tobramycin, gentamicin, and streptomycin [74] have been observed, in vivo tests are not validated for the diagnosis of immediate reactions to aminoglycosides. Moreover, it has been reported that SPT to streptomycin has triggered a systemic reaction [75]. Patch tests with reading at 72 and 96 h have been performed for the diagnosis of non-immediate reactions [76, 77] In vitro tests are not validated. Since aminoglycosides are given by injection, the intravenous route is used for DPT. A graded challenge should be performed only in children with suspected immediate mild reactions. Cross-reactivity between aminoglycosides is common (50%) [1, 78].

### Quinolones

SPT and ID are not recommended for the diagnosis of hypersensitivity to quinolones because they can induce direct mast cells activation, leading to false positive results [1, 79]. Moreover, controversy exists on non-irritant concentrations for skin [1, 79]. In the diagnosis of delayed reactions, patch test has provided inconsistent results [66]. DPT remains the reference standard for the diagnosis even if not without risk [80]. A multidose challenge or a full dose challenge is recommended depending on the clinical risk (Table 3). Cross-reactivity is common between first and second-generation quinolones, less common between first or second generation and third and fourth generation quinolones. Cross-reactions between quinolones and neuromuscular blocking agents have been also described [81].

**Table 3:** Methods of drug provocation test for NBLA

Symptoms	Procedures
Mild urticaria or mild skin reactions	Full-dose challenge
Mild/moderate urticaria or other skin reactions Negative skin tests	Graded challenge (1/100 TD or 1/1000 TD when risk is high, e.g. severe asthma) 1/10 TD 2/10 TD 7/10 TD 1 h observation after each dose
Positive skin tests Anaphylaxis	Desensitization

TD, therapeutic dose

#### DPT to non-betalactam

For NBLAs, specific-IgE measurement methods are not available and other in vitro tests such as basophil activation test (BAT), lymphocyte transformation test (LTT) and ELISPOT test are not routinely used. Furthermore, false positive skin test may occur because nonirritating concentrations are established in adults but not in children [63]. On the other hand, a negative skin test does not exclude the occurrence of an allergic reaction [79]. Despite these limitations, in children with positive NBLA skin test associated with a suggestive history of allergic reaction, the use of alternative antibiotics is recommended. When no alternative antibiotics are available, it is necessary to perform a desensitization [54]. DPT method for NBLA should be based on history and skin tests results (Table 3) [54]. A multidose drug challenge is typically used in children with history of immediate, undefined or delayed reactions. Doses are given orally if possible. It is performed by dividing the therapeutic dose in increasing doses until a single therapeutic dose is obtained. It is generally sufficient to administer 2–3 doses and they can be further divided [4, 5] in case of high risk of reaction. It is suggested not to exceed 5 doses to prevent an unintentional patient desensitization. In patients with mild reaction, a full dose challenge can be performed [54]. In patients with mild/moderate reactions, the starting dose is 1/10 of the therapeutic dose followed by incremental doses (Table 3) or by a full dose [54]. In patients at high-risk because of comorbid disease (e.g. severe asthma), it is suggested to start with 1/100 (or 1/1000) of the therapeutic dose followed by 1/10 and then by incremental doses (Table 3) or a full dose [54]. Doses are commonly given every 30 min, but some authors have recommended a time interval of 1 h [54, 78]. After the last dose, the patient should be monitored for 2–4 h [54]. In children with non-immediate reactions, the drug should be continued at home for 3–5 days and the patient should be instructed to return immediately to the hospital if a delayed clinical reaction occurs [54].

### Non-steroidal anti-inflammatory drugs (NSAIDs)

Ibuprofen, paracetamol and pyrazolones are the most common NSAIDs involved in hypersensitivity reactions [82–84]. NSAIDs can induce both allergic hypersensitivity reactions mediated by an immunological mechanism, i.e. IgE mediated, T-lymphocyte mediated, and non-allergic hypersensitivity reaction initiated by non-immunological mechanisms [24, 27, 77].

*Cross-intolerance hypersensitivity reactions to NSAIDs* are more frequent and include NSAIDs-Exacerbated Respiratory Disease (NERD), NSAIDs-exacerbated cutaneous disease (NECD) (e.g. urticaria), NSAID-induced urticaria-angioedema (NIUA) [27]. They occur up to 6 h after NSAID intake. These reactions are due to a non-immunologic mechanism related to the COX-1 inhibiting properties of the drug that reduces the synthesis of prostaglandins (PG) and thromboxanes, increases the production of leukotrienes (LTs) and induces an overexpression of LTC4 synthase (terminal enzyme for the production of cys-LTs). Although there are two known COX isoenzymes, COX-1 and COX-2, any NSAID that inhibits COX-1, even if belonging to a different class, including aspirin may elicit reaction in susceptible individuals (‘cross-intolerants’) [27, 85]. COX-3, a splice variant of COX-1, has been suggested to be the site of action of paracetamol. This could explain why paracetamol has fewer side effects and less cross-reactions with NSAIDs than other COX inhibitors [86].

*Selective hypersensitivity reactions* to NSAIDs [27] are triggered by a single drug and they rarely may be elicited by more than one NSAID belonging to the same subclass. Other subclasses are tolerated. They include Selective NSAID-induced urticaria, angioedema, and/or anaphylaxis (SNIUAA) and Selective NSAID-induced delayed type hypersensitivity reactions (SNIDR). SNIUAA is probably IgE-mediated reaction occurring within 1 h of the drug intake [85]. Allergic reactions may be of different degrees of severity: from urticaria/angioedema to anaphylaxis. Patients with allergic reactions to an NSAID have had generally at least one prior exposure to the culprit drug, which presumably sensitizes them. Subsequently, the reaction is elicited by following drug exposure. NSAIDs-induced urticaria/angioedema with/without respiratory and systemic symptoms of anaphylaxis (NIUAA) has been also described in children. Selective NSAID-induced delayed type HS reactions (SNIRD) includes a spectrum of skin manifestations such as maculo-papular rash, fixed erythema, contact dermatitis and photoallergy. Sometimes, fever and involvement of internal organs may also occur, while SYS, Lyell Syndrome, and DRESS are rare. These reactions are cell-mediated (Type IV hypersensitivity) and they typically begin 24–48 h after drug intake, but occasionally earlier [27, 87].

### Diagnostic work-up

The diagnostic work-up should be performed 1–6 months after the reaction, the longer the time between the reaction and the skin tests, the lower the negative predictive value and higher the risk of false negatives [88].

SPT, ID, patch test, photopatch test are not standardized [27, 89]. SPT to metamizole, dipyrone, and paracetamol may be helpful in children with immediate reactions [27]. However, their sensitivity is low. Diagnostic accuracy of in vitro tests (serum specific IgE, histamine release test and BAT) has not been established and they are therefore not recommended [27, 90]. Recent studies investigated on BAT as complementary tool for the evaluation of DHRs, revealing a high specificity but a low sensitivity [90]. The diagnostic work-up in DHRs may be expanded and completed with BAT, because the concordance of anamnesis and in vitro tests may reduce the need for challenges, limiting them to selected casesThe diagnosis of NSAID allergy is based on the clinical history and must be confirmed by DPT. DPT is the only commonly available tool to identify the causal role of the suspected drug and it should be performed [86] according to the criteria summarized in Table 1. The negative predictive value of DPT was estimated at about 97.8% [92]. DPT should be also performed to identify a safe alternative drug in subjects with allergy to NSAID [91].

DPT protocols differ in initial dose and time interval between doses. An initial administration of 1/10 of the single therapeutic dose followed by 2/10 and then 7/10 every 60 min has been suggested. Other protocols recommend dividing the therapeutic dose into 3 equal doses, each given at one-hour intervals [93] (Table 4). In patients who have a history of anaphylaxis, the first dose should be 1/100 or 1/1000 of the total cumulative dose [92]. ENDA consider more appropriate performing a multistep challenge, starting with 1/10 of the therapeutic dose also in patients with history of severe reactions or suspected IgE mediated reactions [94]. The total cumulative dose should be: paracetamol 15–20 mg/kg/dose, ibuprofen 10 mg/kg/dose, ASA 15–20 mg/kg/dose [27]. A 3-h observation is recommended after the last dose. In children with history of angioedema, the period of observation should be at least 6 h. In delayed, non-immediate reactions (SNIDRs), longer surveillance period based on history may be necessary. In patients who have experienced wheezing after exposure to an NSAID, spirometry should be performed at the beginning of the DPT and 30 min after each dose. The test should be stopped immediately when a drop in FEV1 ≥ 20% occurs [93]. When a DPT with the culprit drug, has a positive outcome, the question arises whether the reaction is immune-mediated. Patients should undergo an oral challenge to acetylsalycilic acid (ASA) to clarify the mechanism of the reaction. The risk of Reye’s Syndrome is virtually absent, when DPT to ASA is performed in children without infections. A positive DPT indicates that the reaction is non immune-mediated because ASA is a specific inhibitor of COX-1 and a COX-2 inhibitor should be tested [27]. On the other hand, a negative challenge to ASA indicates that the reaction may be immune-mediated and a chemically unrelated NSAID should be tested.

**Table 4:** Provocation test to NSAID in children

	Drug	Dose (mg/kg)	Subsequent doses	Interval between doses (h)	Maximum dose (mg/kg)	Mean reaction time (h)
Kidon MI, 2007 [91]	ASA	2,5	Same dose	1	10	1–4
	Ibuprofen	2,5	Same dose	1	10	2–4
	Paracetamol	5	Same dose	1	20	2–4
Kidon M, 2018 [27]		1/10–1/4 of the maximum dose	Incremental doses	1	- Paracetamol 15–20 mg/kg/dose, -Ibuprofen 10 mg/kg/dose, -ASA 15–20 mg/kg/dose	2–4 Additional doses of the drug can be necessary on the following days at home

## Conclusions

Laboratory tests for identifying children who are allergic to drugs with high diagnostic accuracy and predictive value are not yet available. The provocation test is the gold standard for ascertaining the causative role of an allergen [95, 96], as well as for determining the diagnosis of drug tolerance. However, DPT is time-consuming, resource consuming and may potentially induce serious reactions. Information provided in this paper do not eliminate the need of a proper training to perform this test safely. Therefore, there is an urgent need for the development of simple noninvasive markers [97] for drug allergy in children.

## Abbreviations

AGEP: Generalized acute exanthematous pustulosis
DHR: Drug hypersensitivity reaction
DPT: Drug provocation test
DRESS: Drug eruption with eosinophilia and systemic symptoms
EMM: Erythema multiforme major
ENDA: European Network on Drug Allergy
ID: Intradermal skin test
NBLA: Non-beta-lactam antibiotics
NECD: NSAIDs-exacerbated cutaneous disease
NERD: SAIDs-Exacerbated Respiratory Disease
NIUA: NSAID-induced urticaria-angioedema
SCAR: Severe cutaneous adverse reactions
SJS: Severe skin reactions such as Stevens-Johnson syndrome
SNIRD: Single-NSAID-induced delay and hypersensitivity reactions
SPT: Skin Prick Tests
SSLRs: Serum Sickness-Like Reactions
TD: Therapeutic dose
TEN: Toxic epidermal necrolysis
